# Augmentation index and aortic stiffness in bicuspid aortic valve patients with non-dilated proximal aortas

**DOI:** 10.1186/1471-2261-13-19

**Published:** 2013-03-15

**Authors:** Patrick J Warner, Adeeb Al-Quthami, Erica L Brooks, Alyson Kelley-Hedgepeth, Eshan Patvardhan, Jeffrey T Kuvin, Kevin S Heffernan, Gordon S Huggins

**Affiliations:** 1 MCRI Center for Translational Genomics, Molecular Cardiology Research Institute, Tufts Medical Center, Boston, MA, USA; 2 Division of Cardiology and Vascular Function Study Group, Tufts Medical Center, Boston, MA, USA

**Keywords:** Bicuspid aortic valve, Arterial stiffness, Augmentation index, Pulse wave velocity

## Abstract

**Background:**

We compared aortic stiffness, aortic impedance and pressure from wave reflections in the setting of bicuspid aortic valve (BAV) to the tricuspid aortic valve (TAV) in the absence of proximal aortic dilation. We hypothesized BAV is associated with abnormal arterial stiffness.

**Methods:**

Ten BAV subjects (47 ± 4 years, 6 male) and 13 TAV subjects (46 ± 4 years, 10 male) without significant aortic valve disease were prospectively recruited. Characteristic impedance (Zc) was derived from echocardiographic images and pulse wave Doppler of the left ventricular outflow tract. Applanation tonometry was performed to obtain pulse wave velocity (PWV) at several sites as measures of arterial stiffness and augmentation index (AIx) as a measure of wave reflection.

**Results:**

There were no significant differences between BAV and TAV subjects with regard to heart rate or blood pressure. Zc was similar between BAV and TAV subjects (p=0.25) as was carotid-femoral pulse wave velocity (cf-PWV) and carotid-radial PWV (cr-PWV) between BAV and TAV subjects (p=0.99). Carotid AIx was significantly higher in BAV patients compared with TAV patients (14.3 ± 4.18% versus -3.02 ± 3.96%, p=0.007).

**Conclusions:**

Aortic stiffness and impedance is similar between subjects with BAV and TAV with normal aortic dimensions. The significantly higher carotid AIx in BAV, a proxy of increased pressure from wave reflections, may reflect abnormal vascular function distal to the aorta.

## Background

Bicuspid aortic valve (BAV) is one of the most common congenital cardiac abnormalities, occurring in 1-2% of the population [1]. A high heritability index [2] and association with variants in several genes that regulate heart development [3,4] suggest that BAV is regulated by genetic factors. BAV is also associated with abnormalities of the thoracic aorta including dilation, aneurysm formation, coarctation, and dissection [5,6]. More than half of young patients with normally functioning BAV have echocardiographic evidence of aortic dilatation [7], and progressive aortic dilation may develop in children [8]. The degree of aortic dilation appears out of proportion to valve dysfunction [9,10], and aortic valve replacement does not halt the risk of subsequent aortic complications [7,11]. Finally, aortic aneurysm can present in BAV families as an autosomal dominant trait with incomplete penetrance [12], and data suggests that genetic mutations may contribute to aortic aneurysm in the setting of a BAV [13]. Family studies, however, have not supported shared underlying genetic effects contributing to both BAV and aortic dilation [14]. Thus, evidence would support there being intrinsic abnormalities of aortic stiffness, perhaps caused by genetic factors that may invariably contribute to the risk of dilation and potential for aneurysm formation in BAV.

In this study we prospectively tested our hypothesis by measuring aortic stiffness in a cohort of BAV and TAV patients with normal proximal ascending aorta dimension. In order to further investigate vascular function in BAV, we also measured augmentation index (AIx) as a proxy of pressure from wave reflections and carotid-brachial/ carotid-radial pulse-wave velocity (cb-PWV) as indications of peripheral arterial stiffness.

## Methods

### Subject selection

Study subjects were prospectively recruited from the outpatient echocardiography laboratory at Tufts Medical Center. Valve morphology was assessed using two-dimensional echocardiography in standard long and short axis views. Exclusion criteria were: (i) Marfan syndrome or history of familial aortic aneurysm, (ii) internal ascending aortic diameter greater than 4.5 cm based on echocardiographic measurements, (iii) left ventricular ejection fraction <55%, (iv) moderate or severe aortic stenosis, (v) moderate or severe aortic regurgitation, (vi) aortic valve replacement, (vii) aortic coarctation, and (viii) blood pressure >160/90 mmHg. Ten consecutive BAV subjects (6 males) and 13 TAV controls (10 males) that provided consent were enrolled in the study.

### Ethical clearance and consent

This study was ethically approved by the Tufts Medical Center/Tufts University Institutional Review Board after meeting the required ethical standards in compliance with the Helsinki Declaration. Every prospective participant was explained the purpose of the research and was requested to participate freely. A consent form containing details of the study regarding main purpose, role of participants, and benefits associated with participating in the study (if any) was given to each prospective participant. Those who agreed to participate in the study signed a written consent form. Patients who did not agree to participate in the study were not denied any services or treated with partiality.

### Echocardiography and tonometry measurements

Subjects were studied in the supine position using a tonometry machine (Cardiovascular Engineering, Inc., Norwood, MA) and an echocardiogram machine (Philips IE33 echocardiography machine). Arterial tonometry with ECG was use to record pressure waveforms from the brachial, radial, femoral, and carotid arteries using a custom transducer as previously described [15,16]. Two-dimensional echocardiographic images of the left ventricular outflow tract (LVOT) obtained from the parasternal long axis view were studied to measure the LVOT diameter. Pulsed Doppler of the LVOT from an apical 5-chamber view was recorded to measure LVOT blood velocity.

### Calculation of Pulse Wave Velocity (PWV)

Signal averaged pressure waveforms (NiHem Noninvasive Hemodynamics Workstation v4.50, Cardiovascular Engineering) were superimposed on one another. The body surface distances between the suprasternal notch and pulse recording sites were measured using a measuring tape and calipers. PWV was calculated as the ratio of the distance (Δd) between the carotid artery and a distal arterial site to the relative time interval (Δt) between the foot of the carotid pulse wave and the distal arterial pulse wave (PWV = Δd/ Δt, cm/sec) [15,16]. We directly measured and recorded carotid-femoral PWV (cf-PWV), carotid-brachial PWV (cb-PWV) and carotid-radial PWV (cr-PWV).

### Calculation of augmentation index

The carotid pressure waveform obtained from applanation tonometry was assessed to determine peak pressure (ΔP_total_) and the inflection point created by pressure augmentation of the peripherally reflected wave (ΔP_AI_). The augmentation index (AIx) was computed as the ratio of augmentation pressure to peak pressure expressed as a percentage (AIx = ΔP_AI_/ ΔP_total_). If the inflection point occurs before peak pressure, AIx is expressed as positive percentage; if the inflection point occurs after peak pressure, AIx is expressed as a negative percentage [15]. Time to inflection is presented as a measure of wave travel timing.

*Calculation of Characteristic Impedance:* LVOT absolute flow (ΔV) was calculated as the product of cross sectional area, calculated from the LVOT diameter, and the LVOT blood flow velocity. The pressure differential (ΔP) created during the same time interval was estimated from ECG gated signal averaged pressure waveform acquired from applanation tonometry of the carotid artery. Characteristic impedance (Z_c_) was then calculated as the ratio of pressure to flow (Z_c_=ΔP/ΔV) [15,16].

### Calculation of arterial elastance and peak wall shear

Peak wall shear rate was calculated as 4 × (peak aortic velocity/ aortic root diameter). Cardiac dimensions were assessed using standard 2-dimensional echocardiographic techniques (Simpson’s method). Effective arterial elastance (Ea) was estimated as end systolic pressure / stroke volume and used as a measure of arterial pulsatile load related to vascular input impedance [17]. End systolic pressure was obtained from the carotid pressure waveform. Stroke volume was calculated as end-diastolic volume – end systolic volume from 2D echo.

### Statistical analyses

A priori significance was set at *p* < 0.05. Normality of distribution was confirmed using Kolmogorov-Smirnov and Shapiro-Wilk tests. Group comparisons were made using analysis of variance for continuous variables. Chi-square tests were used to compare categorical variables. All data analysis was carried out using Statistical Package for the Social Sciences (SPSS, v 16.0, SPSS, Inc., Chicago, IL).

## Results

**Figure 1:**
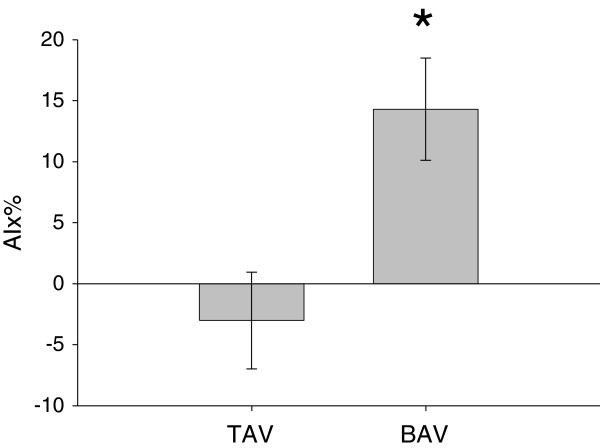
**Subjects with BAV have significantly greater AIx compared with subjects with a TAV.** Carotid AIx was measured by applantation tonometry; AIx was significantly (asterisk, p = 0.007) greater in BAV subjects compared with TAV subjects.

Patient characteristics are listed in Table 1 and show that the two groups were well-matched. Both ascending aorta and aortic root size indexed to body size were similar in both groups. There was a significant difference in aortic peak velocity and wall shear rate in BAV subjects compared to TAV controls (Table 1). A total of 10 BAV patients and 13 TAV controls underwent measures of cb-PWV, cr-PWV and augmentation index. Three patients (1 BAV and 2 TAV) were excluded from the cf-PWV analysis due to poor femoral arterial waveforms. There was no statistical difference in Zc, cf-PWV, cb-PWV or cr-PWV between BAV subjects and TAV controls respectively (Table 2). Similarly, there were no group differences in effective arterial elastance (Table 2). By comparison, BAV participants were found to have significantly larger augmentation indexes compared to TAV controls (Figure 1). This difference in AIx persisted after adjusting for sex, height, and heart rate with analysis of covariance (adjusted means: 14.0% vs −2.8%, p < 0.05). Moreover, adjusting for ascending aortic diameter using a separate model also had no effect on group differences in AIx (adjusted means: 12.5% vs −1.7%, p < 0.05). AIx was significantly correlated with peak aortic velocity (r = 0.45, p = 0.02) and peak wall shear rate (r = 0.49, p < 0.05). Adjusting for either peak aortic velocity (p = 0.11) or peak wall shear rate (p = 0.12) abolished group differences in AIx.

**Table 1:** Demographics

**Descriptives**	**BAV**	**TAV**	**p-value**
Age	46.5 ± 11.6	46.3 ± 15.1	0.97
Males (n, %)	6 (60%)	10 (77%)	0.40
Height (in)	66.0 ± 4.2	70.0 ± 3.5	0.02*
Weight (lbs)	188.9 ± 52.9	180.3 ± 48.9	0.69
Body mass index	30.1 ± 6.8	25.8 ± 5.8	0.10
Body surface area (m^3^)	1.98 ± 0.32	1.99 ± 0.32	0.93
Systolic blood pressure (mmHg)	118.3 ± 14.4	120.7 ± 13.4	0.69
Diastolic blood pressure (mmHg)	71.7 ± 11.6	70.2 ± 7.9	0.72
Pulse pressure (mmHg)	46.6 ± 11.4	50.6 ± 13.0	0.46
Mean arterial pressure (mmHg)	87.2 ± 11.4	87.0 ± 7.9	0.97
Heart rate (bpm)	64.1 ± 6.4	62.4 ± 12.6	0.70
Ejection fraction (%)	59.0 ± 3.1	58.5 ± 3.2	0.67
Aortic root diameter (cm)	3.14 ± 0.52	3.06 ± 0.34	0.75
Aortic root index (cm/m^3^)	1.59 ± 0.18	1.56 ± 0.26	0.69
Ascending aortic diameter (cm)	3.24 ± 0.46	2.87 ± 0.46	0.07
Ascending aortic index (cm/m^3^)	1.67 ± 0.35	1.45 ± 0.20	0.40
Aortic stenosis: mild (n, %)	5 (50%)	None	-
Peak aortic velocity (cm/sec)	217.5 ± 43.3	174.4 ± 45.8	0.001*
Peak wall shear rate (sec^-1^)	285.1 ± 18.9	180.1 ± 20.7	0.001*
Aortic insufficiency:	3 (30%)	2 (15%)	0.02*
Trace (n, %)	4 (40%)	1 (7.7%)	
Mild (n, %)			
**Risk factors (n)**			
Hypertension	5 (50%)	5 (38%)	0.60
Hypercholesterolemia	4 (40%)	6 (46%)	0.85
Coronary artery disease	1 (10%)	2 (15%)	0.20
Tobacco use	1 (10%)	3 (23%)	0.72
Diabetes mellitus	1 (10%)	1 (7.7%)	0.44
**Medications (n)**			
ASA	4 (40%)	2 (15%)	0.72
Anti-coagulants	1 (10%)	2 (15%)	0.20
Beta-blockers	5 (50%)	3 (23%)	0.72
ACE inhibitors/ARB	3 (30%)	3 (23%)	0.42
Statins	3 (30%)	2 (15%)	0.85

Shown are the mean ± standard deviation of the mean.

**Table 2:** Study results

**Arterial Stiffness Parameter**	**BAV**	**TAV**	**p-value**
Characteristic Impedance (Zc)	146.50 ± 14.69	177.54 ± 20.12	0.25
Carotid-Femoral PWV (cm/sec)	781.43 ± 92.51	782.26 ± 67.02	0.99
Carotid-Brachial PWV (cm/sec)	796.14 ± 55.67	789.22 ± 50.44	0.93
Carotid-Radial PWV (cm/sec)	952.65 ± 49.59	940.11 ± 42.64	0.85
Arterial elastance (mmHg/ml)	1.63 ± 0.59	1.94 ± 0.57	0.26
Time to inflection, ms	129 ± 12	141 ± 9	0.39
Augmentation Index (%)	14.27 ± 4.18	-3.02 ± 3.96	0.007*

## Discussion

This study demonstrates that in the setting of a normal ascending aorta size, aortic PWV and arterial elastance appear similar between subjects with BAV and those without. By comparison, BAV participants were found to have a significantly elevated AIx compared to controls. The increased pressure from wave reflections was independent of sex, height and heart rate [18-20]. This finding is clinically significant as increased AIx is associated with increased risk of future CV events [21].

We noted elevated AIx in BAV in the absence of elevated aortic PWV and this is novel. Previous studies noting increased AIx in BAV were carried out in patients with aortic dilation [22,23]. This is important as patients with BAV and dilated aortas have increased aortic PWV compared to BAV patients with preserved aortic size [24]. Dilation of the vessel transposes load bearing from elastin fibers to stiffer collagen fibers, increasing the incremental elastic modulus of the vessel [25,26]. According to the Moens-Korteweg equation, this increase in elastic modulus will increase PWV. Whether increased AIx was secondary to increased aortic stiffness (from altered wave speed) or an independent phenotypic expression of this unique pathology could not be disentangled. Our findings offer important insight into the arteriopathy associated with BAV and suggest increased pressure from wave reflections as a primary systemic vascular aberration in BAV in the absence of aortic dilation, subsequent stiffening and altered reflection timing. These findings suggest a role for downstream microvascular dysfunction distal to the aorta as a potential arbitrator of increased magnitude of pressure from wave reflections in BAV.

A novel observation in the present study was the association between AIx and peak wall shear offering insight into observations of higher AIx in the setting of BAV. BAV patients with normal aortic geometry had higher wall shear than TAV due to higher aortic flow velocity and this is consistent with previous reports noted in BAV patients with dilated aortas and/or aneurysms [27,28]. Altered valve hemodynamics have been implicated in the cause of aortic root dilation [29,30], aneurysm [31] and valve calcification [32,33] in BAV. Such altered central hemodynamics [34] may also damage the peripheral microvasculature [35], altering reflection sites and contributing to increased reflected pressure wave magnitude. It is interesting to note that valve replacement and normalization of aberrant central flow profiles in BAV does not mitigate aortic dilation progression [11]. Abnormalities in AIx reported herein, and elsewhere [22,23], may reflect a previously unsuspected role for increased pressure from wave reflections in the development of large vessel arteriopathy in patients with BAV. In other patient populations such as Marfan syndrome, increased AIx has been shown to predict progression of aortic disease [36,37]. It has been suggested that pressure pulsatility and cyclic stress from wave reflections alter load-bearing capacity of the aortic wall and contribute to fatigue-fracture (i.e. mechanical failure of biomaterials), increasing risk for aortic dilation and aneurysm [38]. The etiology of AIx as it relates to aortopathy in BAV requires further investigation.

The results of our prospective analysis finding no differences in PWV and arterial elastance between BAV and TAV patients is supported by studies showing no difference in serum matrix metalloprotein-2 levels in BAV without aneurysms compared to normal controls [24]. In addition, gene expression profiles of aorta tissue taken from BAV and TAV patients without aneurysm are very similar [39]. Our results differ from studies that demonstrated abnormal aortic root distensibility and stiffness index in BAV patients without aneurysms [40-42]. Discrepancy is likely related to method of measurement as these previous studies relied on imaging modalities such as echocardiography. Our findings are in agreement with others [22,24] demonstrating normal aortic stiffness as measured using PWV in BAV patients without aneurysms. PWV is considered to be a robust measure of aortic stiffness and is currently viewed as the “gold standard” for measuring aortic stiffness [18-20].

We acknowledge several limitations in our study. First, since our primary hypothesis was that abnormal aortic stiffness would be found in the setting of BAV we consider our observation of increased AIx to be hypothesis generating. Second, the small size of our study may limit the generalizability of our results to the broader population of patients with BAV. We noted a partial η^2^ of 0.47 with an observed power of 0.79 signifying moderate effect size with adequate power for detecting group differences in AIx. Thus although possibility of a type II error exists, we believe it to be low.

## Conclusions

Normal aortic stiffness with normal aortic dimension suggests that the ascending aorta is not invariably abnormal in the setting of BAV, despite the predisposition for aorta dilation and aneurysm formation. Our data should motivate further investigation into the impact of the peripheral vasculature on the central vascular phenotype in BAV patients as long term abnormalities may contribute to subsequent ascending aortic dilation and aneurysm formation in patients with BAV.

Our study demonstrates that arterial stiffness and elastance are similar between subjects with BAV and TAV with normal aortic dimensions. At the same time we demonstrate a higher carotid AIx in BAV patients consistent with abnormal vascular properties distal to the aorta. Future studies are required to determine whether abnormal distal arterial properties in BAV subjects contribute to proximal aortic dilation and aneurysm formation.

## Competing interests

The authors declare that they have no competing interests.

## Authors’ contributions

PW recruited and consented subjects for study participation, performed tonometry experiments, assisted in data analysis and manuscript preparation. AAQ directed study implementation, assisted in data analysis and manuscript preparation, ELB directed study implementation, assisted in data analysis and manuscript preparation, AKH directed study implementation, assisted in data analysis and manuscript preparation, EP recruited and consented subjects for study participation, performed tonometry experiments, and assisted in data analysis, JTK oversaw study implementation and assisted in manuscript preparation, KSH directed study implementation, assisted in data analysis and manuscript preparation, and GSH oversaw all aspects of the study including final manuscript preparation. All authors read and approved the final manuscript.

## Pre-publication history

The pre-publication history for this paper can be accessed here:

http://www.biomedcentral.com/1471-2261/13/19/prepub
